# Clinical Features of COVID-19 in Elderly Patients: Tools for Predicting Outcomes Are Needed

**DOI:** 10.3390/jcm11247505

**Published:** 2022-12-18

**Authors:** Riccardo Giorgino, Filippo Migliorini

**Affiliations:** 1 Residency Program in Orthopedics and Traumatology, University of Milan, 20122 Milan, Italy; 2 IRCCS Istituto Ortopedico Galeazzi, 20157 Milan, Italy; 3 Department of Orthopaedic, Trauma, and Reconstructive Surgery, RWTH University Hospital, 52074 Aachen, Germany

The COVID-19 pandemic faced the healthcare landscape with new challenges, impacting work dynamics across all medical disciplines [1,2,3,4]. A prompt and dynamic hospital reorganization was necessary to tackle the pandemic [5,6]. Alongside the management of the COVID-19 pandemic, this reorganization was associated with an improvement in the quality and efficacy of hospital care and services [7]. Hence, the time interval between diagnosis, treatment, and discharge was reduced in several disciplines [8,9]. Following the improvement in hygiene standards, the rate of nosocomial infections was also reduced [9]. 

Given the intrinsic characteristics of COVID-19, elderly patients are particularly at risk, especially those with comorbidities [10,11,12,13]. The limited reserve capacity, fragility and age-related global immune system dysfunction of elderlies, influence the severity and progression of COVID-19 [14,15,16,17,18,19,20]. The identification of patients who are most at risk of complications is essential. Among hospitalized patients, multimorbidity and frailty were highly prevalent [21]. In the geriatric population, acute decompensation of pre-existent comorbidities was the main reason for progression in severity of COVID-19 and longer hospitalization [21]. Multimorbidity also significantly reduced the survival rate of patients infected with COVID-19 [21]. The cause of death in patients infected with COVID-19 was investigated post mortem using clinical chart review and autopsy [22]. Along with hypoxemic respiratory failure, acute decompensation of pre-existent comorbidities within the first week of infection was the most common cause of death [22]. Several studies investigated prognostic factors in elderlies with COVID-19 infections [23,24,25,26,27]. Scheffler et al. investigated the prognostic role of subcutaneous and visceral adipose tissue using a quantification fat area on 64 patients with a mean age of 86.4 ± 6.0 years [23]. There was evidence of a positive association with the subcutaneous and visceral adipose tissue and in-hospital mortality and severe COVID-19 pneumonia [23]. The prognostic value of fever, chest X-ray (CXR), and clinical frailty (CFS) scores were investigated in 122 elderlies aged 65 or older, resulting in these being considered the main predictors of in-hospital mortality [24]. Fever, CXR, and CFS might predict outcomes more accurately than other individual risk factors, confirming the importance of multidimensional assessment of elderlies with COVID-19 [24]. The C_2_HEST has been proposed as a possible tool to evaluate outcomes in elderly patients with active or previous COVID-19 infection. The C_2_HEST is a stratification scoring system to assesses the risk of developing atrial fibrillation. Rola et al. [25] demonstrated that the C_2_HEST was effective in predicting six-month and in-hospital mortality in 1047 elderlies with COVID-19. Moreover, the C_2_HEST was also valid in predicting the risk of non-fatal events, including cardiogenic shock and acute kidney and heart failure in elderlies affected by COVID-19 [25]. COVID-19 negatively impacted the outcomes of elderlies who underwent surgery for lower limb fractures in terms of biochemical parameters (e.g. monocytes, calcium levels, C-reactive protein, creatine phosphokinase, aspartate aminotransferase) and survival [28]. Infection sequelae, including disorientation, fatigue, and dyspnea, might impair postoperative rehabilitation and recovery [28]. Moreover, infected elderlies who underwent surgery for lower limb fractures demonstrated reduced patient-reported outcome measures (PROMs) compared to an age-matched control group of healthy patients [7]. Fericean et al. [29] evaluated whether differences exist in severity progression of infected elderlies between pandemic waves. Among 360 inpatients (60 eligible elderly patients over six consecutive waves) admitted at the Infectious Diseases and Pulmonology Hospital, dyspnea, disorientation, gastrointestinal symptoms, lymphocytosis, and high levels of interleukin-6 were common [29]. Though no significant between waves difference in mortality was reported, a more severe progression of COVID-19 during the third and fourth pandemic waves was observed [29].

In conclusion, given their intrinsic frailty and comorbidities, elderlies are more at risk of a severe COVID-19 progression. The identification of prognostic factors, risk stratification and a tailored management are recommended in this population.
